# Integrating genomic and phenomic data improves prediction of key traits in potato breeding

**DOI:** 10.1093/hr/uhag175

**Published:** 2026-04-30

**Authors:** Alexandre Hild Aono, Rodomiro Ortiz, Aakash Chawade

**Affiliations:** Department of Plant Breeding, Swedish University of Agricultural Sciences (SLU), SE-230 53 Alnarp, Sweden; Department of Plant Breeding, Swedish University of Agricultural Sciences (SLU), SE-230 53 Alnarp, Sweden; Department of Plant Breeding, Swedish University of Agricultural Sciences (SLU), SE-230 53 Alnarp, Sweden

## Abstract

Potato breeding faces challenges due to clonal propagation and complex polyploid genetics, limiting the rate of genetic gain. Predictive breeding integrating genomic and high-throughput phenotypic data offers potential to enhance selection accuracy. Here we evaluated multiple regression models combining genomic single-nucleotide polymorphism (SNP) data and unmanned aerial vehicle-derived image-based traits across diverse potato clones and cultivars to predict key agronomic traits. Combining genomic and phenomic data significantly improved prediction accuracy for total tuber yield compared with using either data source alone, with phenomic data contributing complementary environmental information. Prediction accuracy varied by trait, with genomic data better predicting starch content and phenomic data excelling for tuber yield-related traits. Our findings demonstrate that integrating genomic and phenomic information can enhance predictive breeding strategies, potentially accelerating genetic gain and optimizing resource use in potato breeding programs.

## Introduction

Potato (*Solanum tuberosum*) is a crop of major global importance, providing essential nutrients to the human diet [1]. Increasing potato production is not only crucial for food security but also has significant economic implications, particularly in developing countries, where it serves as an important source of income [2]. Recognized as one of the most impactful agricultural crops in human history [3], potato is currently the most widely cultivated vegetable crop in the world [4].

Potato breeding programs have released a vast number of cultivars, with over 10 000 of them grown globally [5]. Despite these advances, the development of new cultivars remains a pressing need, especially in the face of climate change, growing demand for resilient crops, and the need for broader adaptation across diverse environments [5]. The development of a new potato cultivar typically spans 10–15 years, beginning with a single tuber per clone and progressing through multiple cycles of selection with an increasing number of hills [6]. As a clonally propagated crop with a low propagation coefficient [7], potato breeding faces unique challenges, necessitating innovative strategies to accelerate genetic gain and meet global demands.

High-throughput phenotyping (HTP) offers a promising strategy to overcome key challenges in crop improvement by reducing the labor and costs associated with manual phenotyping while enabling the early identification of superior genotypes [8]. Unmanned aerial vehicles (UAVs) have become valuable tools for field trials, providing efficient crop monitoring that allows breeders to assess environmental effects and evaluate plant responses related to stress tolerance, growth dynamics, and architectural traits [9]. Image-derived data from UAV platforms can be incorporated into predictive breeding models to estimate a wide range of phenotypic traits.

In potatoes, image-based HTP has been applied to diverse traits, including drought response [10, 11], late blight resistance [12–17], insect damage [18], tuber morphology [19], and yield [20, 21]. However, for tuber-related traits such as yield, the relationship between aboveground plant characteristics and belowground tuber performance is often environment-dependent, which complicates the development of robust and transferable prediction models [22].

In addition to HTP, genomic selection (GS) has emerged as a powerful tool for accelerating genetic gains in numerous crops [23]. Several investigations have explored the use of GS in potato breeding, targeting traits such as yield [24, 25], disease resistance [24], and nutritional or quality attributes [25–27]. The literature also reports efforts to enhance GS efficiency in potato through diverse strategies, including the integration of machine learning algorithms for genomic prediction [28], incorporation of family effects into modeling approaches [29], single-nucleotide polymorphism (SNP) subsetting [30], optimization of training populations [31], and resource allocation strategies [32]. Nonetheless, the application of GS in potato remains particularly challenging due to its polysomic tetraploid genome and issues related to self-compatibility [33], which complicate both genomic prediction and marker–trait association analyses.

To overcome these limitations, recent investigations in different crops have studied the integration of GS with HTP, demonstrating that the combination of genomic and phenomic data can enhance prediction accuracy and selection efficiency [34–46]. In potato, image-based data have been used not only to provide more objective trait evaluations but also to enhance genomic prediction of quality traits when integrated with GS [47]. Similarly, the combination of UAV-derived phenotypes with genomic data has shown promise for increasing prediction accuracy. For instance, Maggiorelli *et al.* [48] incorporated mean reflectance from multispectral imagery into genomic prediction models for a range of potato traits, while Yusuf *et al.* [49] applied vegetation indices and other multispectral measures at individual time points to characterize plots and improve predictive performance.

Although these previous studies have demonstrated promising results when integrating multispectral imagery with SNP data for the prediction of several traits in potatoes, replication of individual UAV-derived data points remains challenging due to strong environmental variability. In this context, the area under the curve (AUC) of image-derived traits has been proposed as a more robust indicator of crop growth dynamics [50]. Furthermore, it remains unclear whether similar benefits of integration can be achieved using only red–green–blue (RGB) imagery, an accessible and cost-effective alternative to multispectral platforms.

In this context, our study explores the integration of genomic and UAV-derived RGB traits to improve prediction accuracy in potato breeding. Using a genetically diverse panel of potato breeding clones and cultivars, we evaluated multiple regression-based approaches, including linear mixed-effects models and machine learning algorithms, to predict key agronomic traits from genomic data, phenomic data, and their combination. By leveraging longitudinal UAV data, we summarized vegetation indices across time points using the AUC to capture the dynamic development of potato plants. Our findings highlight the potential of integrating these complementary data sources to enhance selection efficiency and accelerate genetic gains in potato breeding programs.

## Results

### Phenotypic and genotypic data analysis

Evaluation of conventional traits using linear mixed-effects models yielded similar estimates of broad-sense heritability across all traits (Table 1). For total tuber weight, broad-sense heritability estimates were 0.90, 0.70, and 0.90 at Helgegården in 2020 and 2021 and Mosslunda in 2021, respectively. The lowest estimates were observed in Helgegården 2021, with a minimum heritability of 0.63 for the weight of tubers <40 mm. In contrast, the same environment exhibited the highest mean values for total tuber weight and weight across size categories. Among all traits, starch content showed the most consistent heritability estimates across environments.

Image-based traits showed moderate to high broad-sense heritability, ranging from 0.37 for plant height at Helgegården 2021 to 0.80 in Mosslunda 2021 (Table 1). Mean values were generally lower in Mosslunda; for example, the visible atmospherically resistant index (VARI) averaged 0.23 in Mosslunda 2021 compared with 0.39 and 0.61 in Helgegården 2020 and 2021, respectively. Similarly, coverage was 0.29 in Mosslunda 2021 compared with 0.40 and 0.45 in Helgegården. These patterns reflect the trends for tuber weight, with mean values of 6.51 in Mosslunda 2021 versus 10.83 and 14.25 in Helgegården 2020 and 2021. For most traits and environments, the genetic effect was significant, with exceptions including tuber weight <40 and >60 mm in Mosslunda 2021, tuber weight 50–60 mm in Helgegården 2020, starch in Helgegården 2021, and VARI in Mosslunda 2021 (Supplementary Data Table S4).

For all conventional traits, replicate effects were not significant (Supplementary Data Table S5), and row and column effects were significant in only a few cases (*P*-value <0.05), such as total tuber weight in Mosslunda 2021 (Supplementary Data Table S4). In contrast, for image-based traits, significant replicate, row, and column effects were more common, underscoring the greater sensitivity of image-based phenotyping to spatial field variation (Supplementary Data Tables S4 and S5).

**Figure 1:**
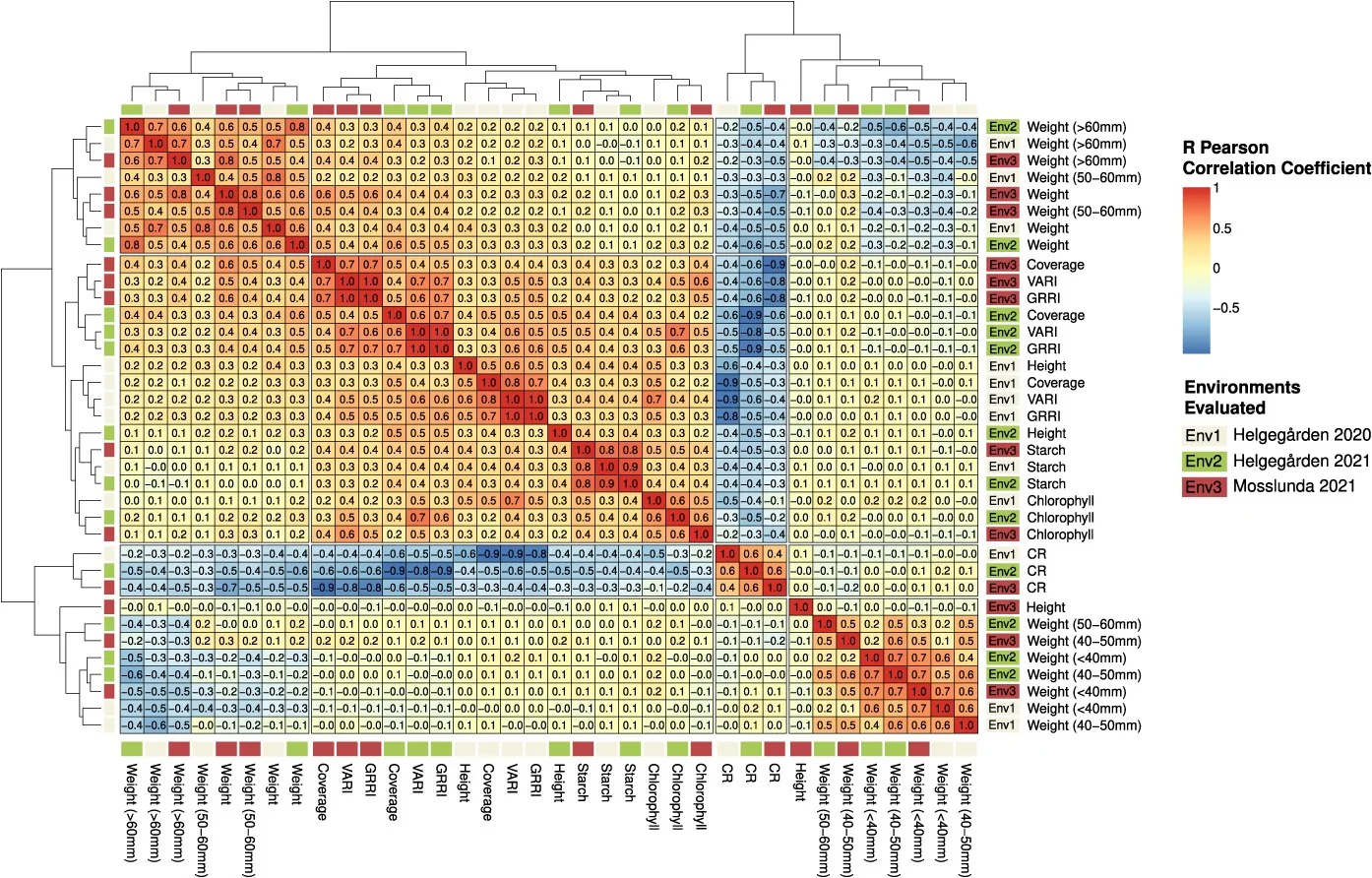
Pairwise Pearson correlations for best linear unbiased estimates (BLUEs) for conventional and image-based traits, separated according to the environment and including: visible atmospherically resistant index (VARI), green–red ratio index (GRRI), canopy reflectance (CR), coverage, plant height, leaf chlorophyll content, total tuber weight, tuber weight by size category (<40, 40–50, 50–60, and >60 mm), and fresh starch content.

Following statistical modeling, best linear unbiased estimates (BLUEs) were calculated for both conventional and image-based traits. Hierarchical clustering of the correlation matrix revealed distinct patterns of trait similarity (Fig. 1). Two main clusters were identified: one included all traits related to tuber weight for smaller size classes (<40 and 40–50 mm), along with canopy reflectance (CR) indices, albeit in a separate subcluster. The remaining traits, except for plant height in Mosslunda 2021 and weight (50–60 mm) in Helgegården 2021, formed a separate group.

Correlations between image-based and conventional traits were highly trait- and environment-specific. For instance, total tuber weight was correlated with coverage and green–red ratio index (GRRI) in Mosslunda 2021 (Pearson’s *R* = 0.6), but in Helgegården 2020 correlations dropped to *R* = 0.3 for both traits. Although some similarities were observed, no consistent general patterns emerged. Typically, the same trait across different environments showed stronger correlations with itself than with other traits. However, exceptions were observed, such as total tuber weight and weight of tubers between 50 and 60 mm.

**Figure 2:**
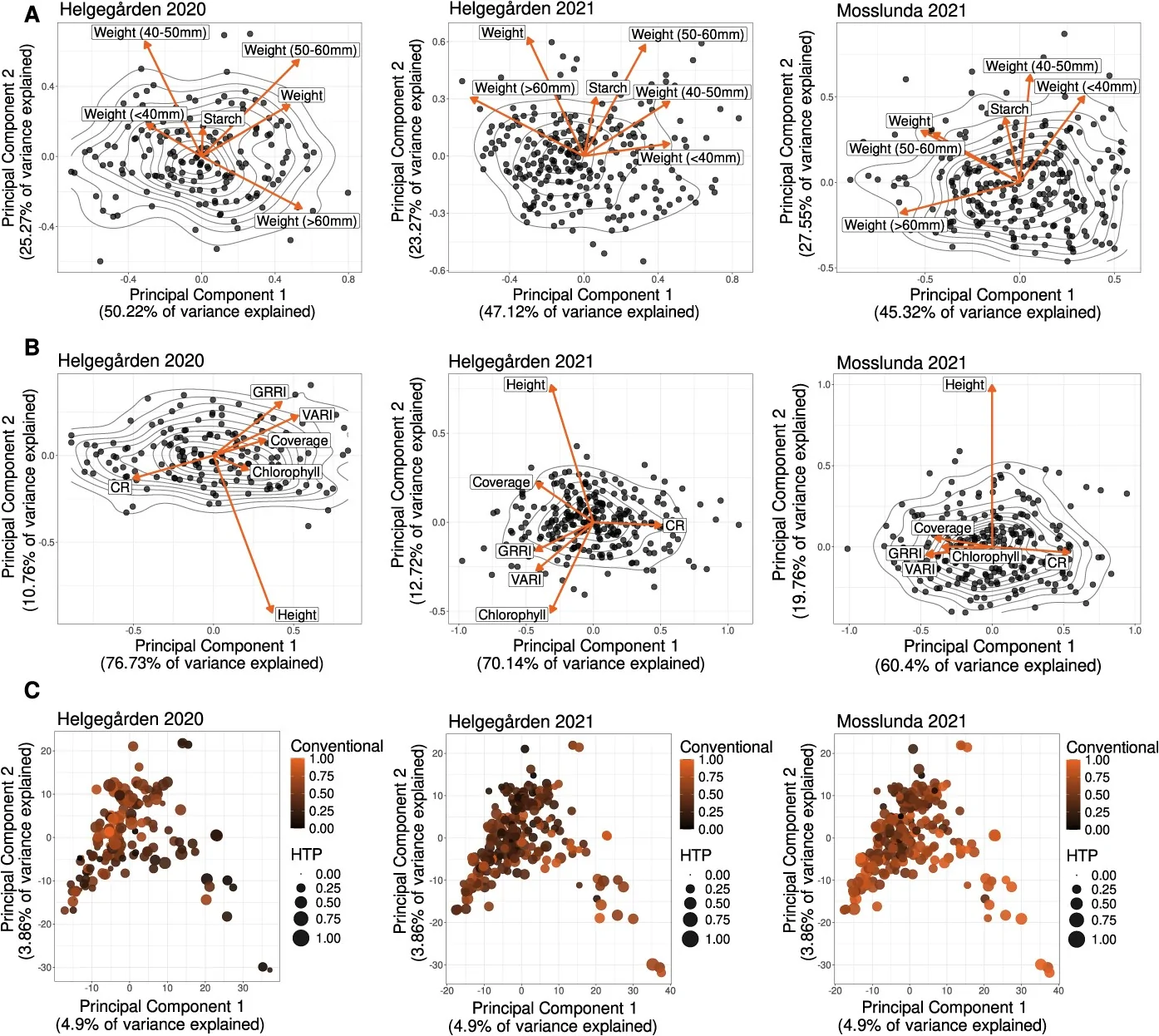
Principal component analysis (PCA) of (A) conventional traits, (B) image-based traits, and (C) genotypic data across the three evaluated environments. For phenotypic traits, the values used in the PCA were the best linear unbiased estimates (BLUEs) derived from linear mixed-effects models. The traits included visible atmospherically resistant index (VARI), green–red ratio index (GRRI), canopy reflectance (CR), canopy coverage, plant height, leaf chlorophyll content, total tuber weight, tuber weight by size category (<40, 40–50, 50–60, and >60 mm), and fresh starch content. In each panel, individual points represent different genotypes. In panel (C) the PCA was performed using genome-wide marker data. The color of each point represents the first principal component (PC1) derived from conventional traits (panel A), whereas point size corresponds to the first principal component (PC1) derived from image-based traits (panel B). Arrows in panels (A) and (B) indicate the projection of each trait into the principal component space, representing their direction and contribution to the total variance.

Principal component analysis (PCA) on conventional (Fig. 2A) and image-based (Fig. 2B) traits indicated similar variance trends across environments. No strong clustering of arrows (traits) was observed, suggesting limited multicollinearity among traits. For conventional traits, contributions to PC1 and PC2 were relatively balanced. In contrast, for image-based traits plant height contributed more strongly to PC2 than to PC1, indicating that potentially the variance associated with plant height was distinct from other image-based traits.

This integrated visualization allows assessment of the correspondence between genetic structure and phenotypic variation, highlighting whether major axes of phenotypic differentiation align with underlying genomic relationships.

The PCA of genotypic data (Fig. 2C) revealed no clear population structure, though a few individuals appeared slightly more divergent. However, the variance explained by the first principal components was low (<10%). When the genotypic PCA was colored and scaled according to the phenotypic principal components, no clear correspondence between genotype and phenotype was observed, apart from noticeable differences in distribution patterns across environments.

### Genomic prediction models

The first set of regression models developed were genomic prediction models aimed at predicting the BLUEs of conventional traits (Supplementary Data Table S6). Using both CV1 and CV00 cross-validation (CV) strategies within each environment and multiple modeling approaches, overall model performance was generally low, underscoring the challenge of accurately predicting the performance of untested genotypes. Starch content was the only trait for which prediction accuracy exceeded an *R*^2^ of 0.5.

For starch content, similar accuracies (*R*^2^ = 0.05) were obtained using both CV00 and CV1, likely reflecting the strong correlations between starch BLUEs across environments, with Pearson *R* correlation coefficients exceeding 0.8. A comparable, though less pronounced, pattern was observed for tuber weight in the >60 mm size class, with lower cross-environment correlations (*R* > 0.6).

For tuber weight in the <40 mm (*R*^2^ = 0.4) and 40–50 mm (*R*^2^ = 0.2) size classes, similar model performance was also observed between CV1 and CV00 in Helgegården 2020 and 2021. In contrast, for total tuber weight and the 50–60 mm size class, prediction accuracies remained low in Helgegården 2020 and 2021 (*R*^2^ < 0.2). For these traits, the highest accuracies were obtained when predicting Mosslunda 2021 BLUEs, reaching an *R*^2^ of ~0.4 for total tuber weight in CV1.

Across all traits and environments, differences among modeling strategies were minimal. One exception was the multilayer perceptron (MLP) neural network, which consistently yielded the lowest accuracies. This underperformance likely reflects the sensitivity of neural networks to hyperparameter tuning. In this study, no specific hyperparameter optimization was conducted, which may have limited the MLP’s predictive ability.

When considering the highest prediction accuracy for each environment–trait combination under the CV1 strategy and conventional traits, Bayesian ridge regression (BRR) achieved the best performance in 11 combinations, followed by genomic best linear unbiased prediction (G-BLUP) (5 combinations) and machine learning methods (4 combinations) (Table 2). The highest observed prediction accuracies ranged from an *R*^2^ of 0.117 (for tuber weight in the 50–60 mm class in Helgegården 2021) to 0.511 (for starch content in Helgegården 2021). Overall, Mosslunda 2021 yielded the highest prediction accuracies, potentially reflecting differences in management practices at this location.

**Table 1:** Broad-sense heritability (*h*^2^) estimates for conventional and image-based traits across environments (two locations and two years). Values are presented as *h*^2^ (trait mean).

**Trait Category**	**Trait**	**Helgegården 2020**	**Helgegården 2021**	**Mosslunda 2021**
Conventional Traits	Weight	0.90 (10.83)	0.70 (14.25)	0.90 (6.51)
	Weight (>60 mm)	0.90 (3.27)	0.75 (7.90)	0.84 (1.94)
	Weight (50–60 mm)	0.83 (4.13)	0.72 (4.22)	0.82 (2.34)
	Weight (40–50 mm)	0.87 (2.79)	0.72 (1.77)	0.79 (1.80)
	Weight (<40 mm)	0.92 (0.63)	0.63 (0.39)	0.81 (0.44)
	Starch	0.94 (14.96)	0.85 (14.48)	0.90 (13.42)
Image-based traits	VARI	0.62 (0.39)	0.65 (0.61)	0.68 (0.23)
	GRRI	0.59 (0.64)	0.62 (0.59)	0.67 (0.25)
	CR	0.56 (0.68)	0.73 (0.43)	0.71 (0.18)
	Coverage	0.45 (0.45)	0.66 (0.40)	0.58 (0.29)
	Chlorophyll	0.79 (0.38)	0.58 (0.33)	0.54 (0.05)
	Height	0.53 (0.62)	0.37 (0.62)	0.80 (0.22)

In addition to conventional traits, we assessed the feasibility of genomic prediction for image-based traits (Supplementary Data Table S7). Although these traits exhibited heritability patterns comparable to those of conventional traits (Table 1), their prediction accuracies were lower, with no trait achieving *R*^2^ > 0.25.

As observed for conventional traits, the performance of different genomic prediction methods for image-based traits was generally similar. Given the overall low accuracies, no consistent differences were observed between the CV1 and CV00 strategies. When evaluating the highest *R*^2^ values for each trait–environment combination under CV1 (Table 2), G-BLUP showed the best performance in 10 combinations, followed by BRR in 8 combinations and machine learning methods in 4 combinations.

### Phenomic prediction models

In addition to genomic prediction models, we evaluated the use of image-based (phenomic) traits to predict the BLUEs of conventional traits. Overall, genomic prediction models were more accurate than phenomic prediction models, with the exception of total tuber weight (Supplementary Data Table S6). Under the CV1 strategy, the best prediction accuracies for total tuber weight ranged from an *R*^2^ of 0.191 in Helgegården 2020 to 0.429 in Mosslunda 2021 (Table 1). Compared with genomic prediction, these accuracies represented improvements of ~22% in Helgegården 2020, 200% in Helgegården 2021, and 8% in Mosslunda 2021.

For phenomic prediction, CV1 generally outperformed CV00 (Supplementary Data Table S6), except for starch content, where the highest accuracies (*R*^2^ = 0.2) were observed for predictions in Mosslunda 2021, regardless of the training environment. For tuber weight across different size categories, the highest accuracies were also achieved under CV1, particularly for tubers >60 mm in Helgegården 2021 and Mosslunda 2021 (both *R*^2^ = 0.201). For all other size categories, *R*^2^ values remained <0.2.

As with genomic prediction, the performance of different modeling strategies in phenomic prediction was generally comparable. When identifying the best-performing model for each trait–environment combination (Table 1), BRR achieved the highest accuracy in 10 combinations, followed by machine learning methods in 8 combinations, and phenomic best linear unbiased prediction (P-BLUP) in 2 combinations. Notably, the best-performing machine learning model varied across traits and environments, with each algorithm tested performing best in at least one of the eight combinations where machine learning methods achieved the highest accuracy.

### Combining strategies

To combine genomic and phenomic data, we adopted a kernel-based regression strategy, assigning a kernel to each omics layer and integrating them into a single model (G + P-BLUP). We selected this approach because datasets with large numbers of SNPs or image-based traits can affect the performance of feature-based regression models. Given that increasing the number of features leads to reduced changes in kernel structures, we considered that comparing the results obtained with G-BLUP, P-BLUP, and G + P-BLUP could provide insights relevant to other studies and datasets.

Initial comparisons were conducted using a leave-one-out (LOO) CV strategy (Supplementary Data Table S8). Overall, G + P-BLUP performed best under the CV1 strategy, with modest improvements also observed for CV00 in some trait-environment combinations. Based on the CV1 results, traits could be grouped into two categories. Starch content and the weight of small tubers (<40 and 40–50 mm) showed the best performance with G-BLUP alone. In contrast, total tuber weight and the weight of larger tubers (50–60 and >60 mm) consistently achieved the highest accuracies with G + P-BLUP across all environments. This pattern was consistent with the trait groups exhibiting higher correlations between BLUEs (Fig. 1).

**Figure 3:**
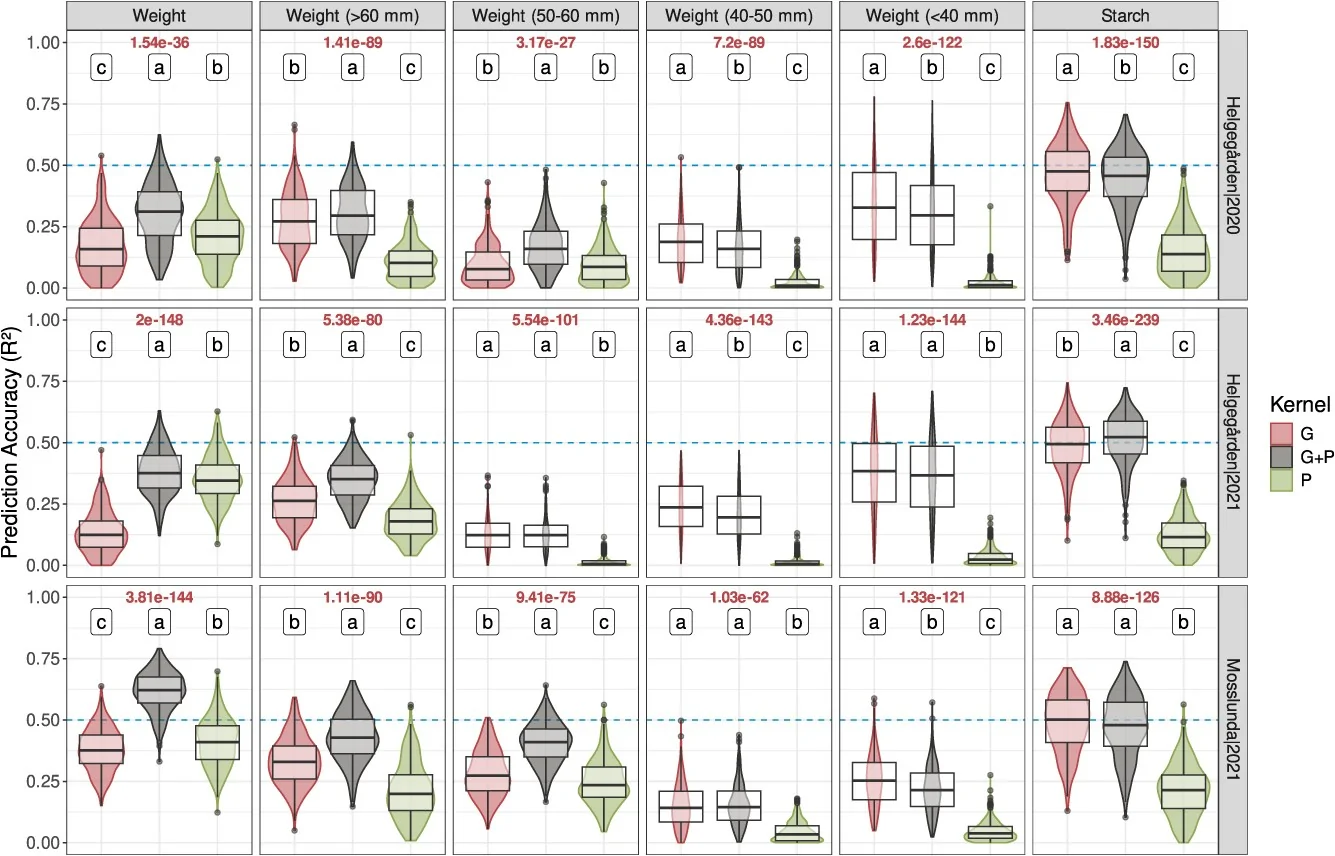
Prediction accuracy of kernel-based regression models using genomic (G), phenomic (P), and combined (G + P) data sources in a 5-fold cross-validation scheme (CV1). Accuracy is measured as the squared Pearson correlation coefficient (*R*^2^) and presented as boxplots. The Kruskal–Wallis rank-sum test was used to assess differences among data sources for each trait–environment combination, with corresponding *P*-values shown in red. When significant differences were detected, pairwise Wilcoxon rank-sum tests were performed to identify the top-performing data source(s), indicated by labels next to the boxplots.

When the same comparisons were repeated using a 5-fold CV scheme (Supplementary Data Table S9), similar results to those observed under LOO CV were obtained. Specifically, G + P-BLUP significantly outperformed G-BLUP and P-BLUP for total tuber weight and the weight of larger tubers (50–60 and >60 mm), as shown in Fig. 3. The only exception was Helgegården 2021, where no significant difference was observed between G-BLUP and G + P-BLUP for tuber weight in the 50–60 mm size class.

Conversely, for starch content and small tuber weight (<40 and 40–50 mm), genomic prediction alone generally yielded significantly better or comparable results than the integration of both omics sources.

For total tuber weight and weight of tubers >60 mm, G + P-BLUP achieved prediction accuracies exceeding *R*^2^ = 0.3, corresponding to a Pearson correlation coefficient of ~0.55. This represents a roughly 2-fold improvement over the performance of G-BLUP alone. In cases where phenomic data did not contribute to prediction, such as the weight of small tubers (<40 and 40–50 mm), the inclusion of phenomic information did not negatively impact model performance.

**Figure 4:**
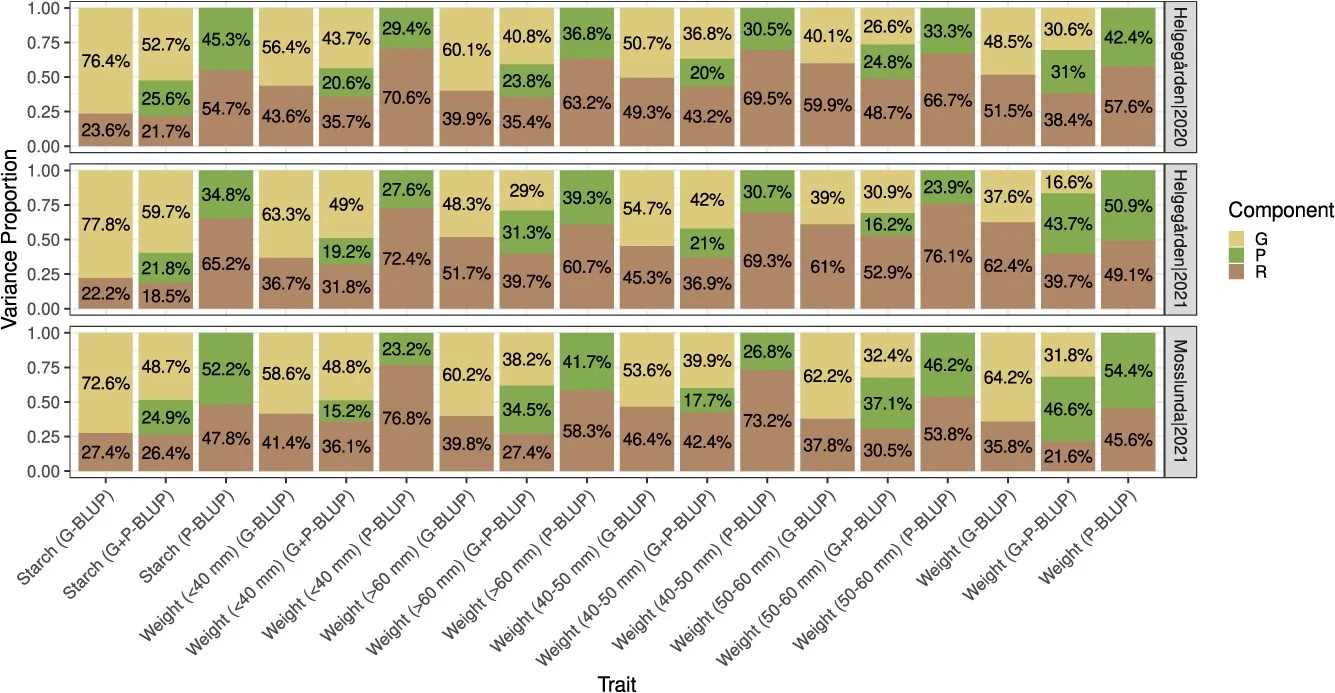
Percentage of variance explained by the genomic (G), phenomic (P), and residual (R) components in the kernel-based best linear unbiased prediction (BLUP) models (G-BLUP, P-BLUP, and G + P-BLUP). For each combination of trait, environment, and model, the stacked bar plots show the proportion of variance attributed to each random effect.

To investigate the complementary nature of the data sources, we evaluated the percentage of variance explained by each component in the models (Fig. 4.). Similar to the pattern observed for the two trait groups (starch content and small tuber weight versus total tuber weight and weight of larger tubers), the proportions of explained variance showed a consistent pattern within each group but differed between groups.

For starch content and small tuber weight, the inclusion of phenomic information added little to the variance already explained by genomics. Instead, phenomics appeared to capture variation that largely overlapped with the genomic component. For example, for starch content in Helgegården 2020, the genomic component in the G-BLUP model explained ~76.4% of the total variance. When using the G + P-BLUP model, the combined genomic and phenomic components explained ~78.3%. Similar trends were observed across the other environments and traits, with only minor reductions in residual variance.

In contrast, for total tuber weight and the weight of larger tubers, the inclusion of phenomic information substantially reduced the residual variance, indicating a complementary contribution. For total tuber weight in Helgegården 2020, the residual variance decreased from ~51.5% in the G-BLUP model to 38.4% in the G + P-BLUP model. In Helgegården 2021 and Mosslunda 2021 the residual variance was reduced from ~62.4% to 39.7% and from 35.8% to 21.6%, respectively. The other traits within this group showed a similar pattern.

## Discussion

Predictive breeding approaches based on genomics and phenomics represent a viable alternative to conventional phenotypic selection for increasing genetic gains in potato breeding programs. Given the clonal propagation system and the extensive field trials required to develop new varieties, accelerating genetic improvement in potato remains a major challenge [5, 7]. However, the complexity of polyploid genetics and the specific characteristics of the crop pose significant challenges to the development and implementation of predictive models. Across the literature, genomic prediction models for highly quantitative traits in potato have demonstrated only low to moderate accuracy [27, 51]. Consistently, in our study all evaluated traits showed prediction accuracies <0.5 under CV1 and CV00 cross-validation schemes.

In the context of phenomic prediction, the underground nature of tuber traits makes them particularly difficult to predict using aboveground image-based data, especially given their strong environmental dependency [22]. Our results corroborate this challenge, as the associations between image-based and conventional traits were highly environment-specific. While phenomic prediction achieved comparable or slightly better accuracies than genomic prediction for total yield, it underperformed for other tuber-related traits.

Interestingly, image-based traits revealed subtle yet consistent differences across replicates, rows, and columns, which were effectively accounted for by the mixed models used to estimate BLUEs. In contrast, such spatial variation was less evident for conventional traits, suggesting that image-derived traits are more sensitive to genotype-specific physiological responses to micro-environmental heterogeneity. Consistent with this interpretation, genomic prediction models for image-based traits exhibited lower accuracies than those for conventional traits, indicating a weaker direct association between image-derived phenotypes and underlying genomic variation. Image-based traits reflect dynamic physiological states measured across time, which are shaped by complex processes extending beyond genetic control, including environmental modulation and temporal plasticity [52].

If we conceptualize phenotypes as the result of interactions across multiple molecular layers, such as the genome, transcriptome, and proteome, then environmental signals play a crucial role in modulating these interactions rather than altering quantitative trait loci (QTLs) [53]. In this light, image-based traits can provide valuable insights into plant physiological status and stress responses [54], indirectly reflecting the modulation of molecular networks under specific environmental conditions. When integrated with genomic data, image-based information can enhance the prediction of breeding values by capturing both genetically determined and environmentally induced phenotypic variation. Because our analyses were conducted within single environments without explicitly modeling genotype-by-environment interactions, the resulting BLUEs likely retained an environmental component that could not be fully captured by genomic information alone. This was particularly evident in our results for total weight and weight by size categories (Fig. 3), where combining data sources increased prediction accuracy and revealed complementary contributions to trait variance (Fig. 4.).

However, this pattern was not consistent across all traits. The prediction of BLUEs for starch content and weight of small tubers did not benefit from the inclusion of image-based data. For starch content, previous studies have shown that this trait may exhibit relatively low variability across environments in certain populations [55]. Consistently, we observed strong correlations of starch BLUEs across environments (Fig. 1). We therefore hypothesize that image-derived phenomic information did not substantially improve prediction because the reduced environmental sensitivity of the trait limited the contribution of phenomics in capturing additional environmental variation. Moreover, the prediction of nutritional traits using drone-based imagery has been reported to be less accurate than that of yield-related traits [48], possibly due to the weaker association between nutritional composition and aboveground phenotypes. At the same time, starch showed the highest genomic prediction accuracies (Fig. 3), suggesting a comparatively simpler genetic architecture relative to total tuber weight, which is strongly influenced by environmental fluctuations [56].

For the weight of small tubers, the limited contribution of phenomics, and the skewed distribution of genomic prediction accuracies (Fig. 3) may be explained by the distributional properties of the trait itself. Small tubers represent the lowest proportion of both tuber number and total tuber weight. Consequently, most genotypes exhibit values close to zero, resulting in a skewed distribution, which makes the trait more difficult to predict accurately or associate with genomic information.

Although omics technologies can provide detailed molecular profiles of traits, their practical use in breeding remains limited by high cost and technical complexity. In contrast, UAV-based image acquisition adds minimal cost to traditional field phenotyping [57]. The main constraint, however, is that image data can only be collected once the plants are grown, whereas genomic data can be obtained from seedlings, thereby reducing field trial costs. Yet, in our study genomic prediction alone yielded modest accuracies, underscoring its limitations.

Different from previous attempts to integrate genomics and phenomics in potatoes [47, 48], we have employed a cheaper image-acquisition methodology, relying on RGB imagery instead of multispectral information. Even with reduced information, we were able to see improvements in the combination of both sources, corroborating the potential of introducing phenomics into potato breeding. Additionally, employing AUC measurements was shown to be promising in our study, not only showing improvements in prediction, but also moderate to high values of accuracy across image-based traits. We believe that, by doing so, our approach can be extended to other scenarios, even including data points different from the ones evaluated in our study.

As observed in previous studies, altering the modeling approach in genomic prediction often yields only marginal gains in accuracy [58, 59]. Similarly, in our analyses the best-performing regression model varied across trait–environment combinations, with no single model outperforming others substantially. Given the robust performance of G-BLUP in handling high-dimensional SNP data [60], we selected this method as a baseline for comparison with models that integrated image-based traits. These combined models utilized a kernel-based strategy that incorporates image-derived similarity matrices, which we interpret as reflecting the integrated physiological state of plants.

Kernel-based approaches are particularly advantageous for modeling image-based traits, as they do not require prior trait selection. This flexibility enables the incorporation of additional image information, including features extracted from deep learning models [61], without substantially altering the modeling framework. Additionally, this strategy allows a more balanced integration of different omics layers, where each layer contributes independently rather than through predefined covariates. Our variance partitioning results support this interpretation.

In terms of practical breeding outcomes, our findings indicate that combining genomics and image-based traits can improve yield predictions, a central selection target in potato breeding programs [7]. However, results were less promising for starch content and the weight of small tuber sizes. For such traits, other HTP technologies, such as near-infrared spectroscopy (NIRS), may offer better predictive value [62]. Nonetheless, practical implementation requires careful field design to optimize the trade-off between cost and genetic gain.

In the early stages of potato breeding (e.g. single-hill or early clonal generations), the large number of genotypes makes GS costly. At this phase, we recommend the use of HTP data collected at multiple time points to capture growth and development dynamics. Phenomic prediction can serve as a filter for low-performing clones before committing to more expensive genotypic or labor-intensive phenotypic assays. Our results indicate that this strategy is not only a cost-effective early selection tool, but also improves trait modeling and breeding value estimation.

As the program progresses to intermediate cycles and the number of genotypes is reduced, GS becomes more attractive. If image data were collected in earlier cycles, integrating phenomic and genomic data would be both practical and advantageous, as demonstrated by the improved prediction accuracies observed in our study. If such data are not available, GS could still be implemented, with performance depending on the composition and size of the training population. Over time, as training populations grow, the accuracy of genomic predictions improves, and genetic gains are expected to surpass those of conventional phenotypic selection, as shown in previous studies [32].

Once the program reaches the later stages, genotypic information becomes available for all individuals, enabling the full implementation of integrated genomics–phenomics models. As shown here, these models enhance predictive performance. Moreover, because HTP data can capture plant responses to environmental variation and stress [8], their inclusion can support indirect selection for stress resilience. Considering that multi-environment field trials are typically expanded at this stage, integrating phenomic and genomic data can further improve the accuracy of selecting the most promising genotypes.

Looking forward, the increasing adoption of Internet of Things (IoT) technologies, field sensors, robotics, and artificial intelligence will likely make the integration of genomic and phenomic information more accessible and impactful [63]. In this context, breeding strategies that combine both data sources are likely to become increasingly influential in shaping future crop improvement strategies.

### Conclusions

In our study, we hypothesized that imagery-based characterization provides indirect yet biologically meaningful insights into the modulation of molecular networks under specific environmental conditions. When integrated with genomic information, these signals offer a more comprehensive understanding of phenotypic variation. Our kernel-based modeling framework successfully integrated multiple omics layers, demonstrating that the joint use of genomic and phenomic data improves predictive performance, particularly for tuber yield, a primary breeding target. Given that potato is clonally propagated and breeding schemes involve evaluating multiple clones per genotype across stages, the incorporation of high-throughput phenotyping alongside genomics provides complementary and cost-effective information to enhance selection decisions throughout the breeding pipeline.

Importantly, UAV-based image acquisition adds minimal cost to conventional field phenotyping workflows. We further demonstrated that low-cost RGB imagery alone was sufficient to improve genomic prediction for yield. By summarizing temporal dynamics using the AUC, our approach avoids dependence on isolated time points and instead captures the integrated developmental trajectory of the crop. This strategy supports transferability across environments, populations, and analytical frameworks.

## Materials and methods

### Plant material and conventional phenotyping

The plant material consisted of 256 potato breeding clones and cultivars (*Solanum tuberosum* L.; Supplementary Data Table S1), evaluated in incomplete block designs across two experimental locations, Helgegården and Mosslunda (details in Table 3). At Helgegården trials were conducted in 2020 and 2021, whereas at Mosslunda evaluations were carried out only in 2021. Both sites are situated in rural areas near Kristianstad, Sweden. Each plot consisted of 10 plants spaced 0.3 m apart within rows, with 0.75 m between rows. Mosslunda was designated as a disease evaluation site, and therefore no fungicides were applied during the growing season. In contrast, at Helgegården fungicides were applied to control *Phytophthora infestans* and prevent potato late blight. For analysis, each location–year combination was considered an independent environment.

**Table 2:** Prediction accuracies (squared Pearson correlation coefficients, *R*^2^) for the top-performing models used to predict BLUEs of conventional and image-based traits in a LOO cross-validation approach (CV1). Models were trained using either genomic or phenomic data as input features.

**Trait**	**Helgegården 2020 Genomics**	**Phenomics**	**Helgegården 2021 Genomics**	**Phenomics**	**Mosslunda 2021 Genomics**	**Phenomics**
Weight	0.156 (BRR)	0.191 (BRR)	0.123 (BRR)	0.386 (BRR)	0.396/0.397 (G-BLUP/BRR)	0.429 (BRR)
Weight (>60 mm)	0.279 (G-BLUP)	0.074 (P-BLUP)	0.288 (RF)	0.201 (BRR)	0.335 (BRR)	0.201/0.191 (P-BLUP/SVM)
Weight (50–60 mm)	0.122/0.219 (RF/AB)	0.093 (BRR)	0.117 (RF)	0.013 (MLP)	0.284 (BRR)	0.019 (RF)
Weight (40–50 mm)	0.173 (RF)	0.008/0 (RF/MLP)	0.225 (G-BLUP)	0.009 (AB)	0.143/0.144 (G-BLUP/BRR)	0.054 (BRR)
Weight (<40 mm)	0.315 (BRR)	0.143 (BRR)	0.379 (BRR)	0.014 (MLP)	0.245 (BRR)	0.184 (BRR)
Starch	0.483 (BRR)	0.162/0.164 (BRR/SVM)	0.511 (BRR)	0.16 (BRR)	0.504 (G-BLUP)	0.209 (AB)
VARI	0.128 (G-BLUP)		0.187 (G-BLUP)		0.2 (G-BLUP/BRR)	
GRRI	0.096 (G-BLUP/BRR/RF)		0.149 (BRR)		0.218 (BRR)	
CR	0.055 (RF)		0.185 (G-BLUP)		0.176 (BRR)	
Coverage	0.065 (RF)		0.164 (G-BLUP)		0.18 (BRR)	
Chlorophyll	0.228 (G-BLUP)		0.226 (G-BLUP)		0.139 (BRR)	
Height	0.068 (G-BLUP)		0.064 (G-BLUP/BRR)		0.007 (SVM)	

**Table 3:** Experimental design in each site.

**Location (coordinates)**	**Year**	**Genotypes**	**Repetitions**	**Experimental Design**
Helgegården (56°00′56″N 14°03′50″E)	2020	169	2	13 × 13 simple lattice
	2021	256	2	16 × 16 simple lattice
Mosslunda (55°58′04″N 14°06′51″E)	2021	256	2	16 × 16 simple lattice

The potato growing season in both locations typically spans from late May to early September (~3.5–4 months). Across the cropping seasons of 2020 and 2021, average monthly temperatures differed by <1°C, except in July and August, which showed deviations of ~4 and 2°C, respectively (Supplementary Data Table S2).

Conventional traits measured included total tuber weight and tuber weight by size category (<40, 40–50, 50–60, and >60 mm), all recorded in kilograms per plot. In addition, tuber fresh starch content was assessed as a percentage, estimated via specific gravity measurements obtained after harvest.

### High-throughput phenotyping

In addition to conventional traits, aerial imagery was collected for the field trials using a DJI Mavic 2 Pro UAV, equipped with an integrated 20-MP RGB camera. A front and side overlap of 75% was set to ensure complete coverage and facilitate high-quality image orthomosaicking. Flights were conducted at an altitude of 20 m above ground level. For the Helgegården 2020 field trial, imagery was captured on 8, 22, and 31 July, as well as 2, 10, and 16 September. For Helgegården 2021, data were collected on 7 and 20 July, 4 and 24 August, and 9 September. In Mosslunda 2021, flights were performed on 28, July 7 and 20 June and 4 and 24 August.

Processing of images from each flight, including orthomosaics, plot boundaries, and trait extraction, was done using the software Pysel (Phenoyard AB, Sweden). For each plot, the following vegetation indices and plant measurements were computed (Supplementary Data Table S3): VARI, GRRI, CR, coverage, plant height, and leaf chlorophyll content. For each plot, VARI and GRRI values were averaged per image. The final image-based traits of each plot were computed using the AUC method across all timepoints. AUC was calculated in R statistical software v4.1.2 [64] using the trapezoidal rule [65]:


(1) \begin{equation*} AUC=\sum \limits_{i=\mathrm{1}}^{n-1}\frac{T\left({t}_i\right)+T\left({t}_{i+1}\right)}{2}\times \left({t}_{i+1}-{t}_i\right), \end{equation*}

where *T*(*t_i_*) represents the trait value at timepoint *t_i_* (*t*_1_, *t*_2_, … *t_n_*).

### Phenotypic data analyses

Conventional and image-based traits were analysed using linear mixed-effects models implemented in the ASReml-R package v4.2.0.355 [66] together with R statistical software v4.1.2 [64]. Variance components were estimated using the restricted maximum likelihood (REML) approach. For each environment (i.e., the combination of location and year), the following model was applied:


(2) \begin{equation*} {y}_{ijkl}=\mu +{r}_j+{p}_{jk}+{c}_{jl}+{g}_i+{\epsilon}_{ijkl}, \end{equation*}

where *y_ijkl_* is the observed trait value of the *i*th genotype in the *j*th replicate, located in the *k*th row and *l*th column; *μ* is the fixed overall mean; *r_j_* is the fixed effect of the *j*th replicate; *p_jk_* is the random effect of the *k*th row within the *j*th replicate (${p}_{jk}\sim N\mathrm{\big(0,}{\sigma}_p^2\big)$, where ${\sigma}_p^2$ is the variance of the row-by-replicate interaction); *c_jl_* is the random effect of the *l*th column within the *j*th replicate (${c}_{jl}\sim N\mathrm{\big(0,}{\sigma}_c^2\big)$, where ${\sigma}_c^2$ is the variance of the column-by-replicate interaction); *g_i_* is the random effect of the *i*th genotype (${g}_i\sim N\mathrm{\big(0,}{\sigma}_g^2\big)$, where ${\sigma}_g^2$ is the genetic variance); and ${\epsilon}_{ijkl}$ is the residual error (${\epsilon}_{ijkl}\sim N\mathrm{\big(0,}{\sigma}_{\epsilon}^2\big)$, where ${\sigma}_{\epsilon}^2$ is the residual variance). All random effects were assumed to be independent and identically distributed.

The significance of fixed effects was determined using Wald *F*-tests. The significance of random effects was assessed through likelihood ratio tests (LRTs) via analyses of deviance (ANODEV). Broad-sense heritability (*h*^2^) was calculated according to Piepho and Möhring [67]:


(3) \begin{equation*} {h}^2=\frac{\sigma_g^2}{\sigma_g^2+\frac{\sigma_p^2}{r}+\frac{\sigma_c^2}{r}+\frac{\sigma_{\epsilon}^2}{r}}, \end{equation*}

where ${\sigma}_g^2$, ${\sigma}_p^2$, ${\sigma}_c^2$, and ${\sigma}_{\epsilon}^2$ represent the genetic, row-within-replicate, column-within-replicate, and residual variances, respectively; and *r* is the number of replicates.

For subsequent analyses, genotype effects (*g*) were treated as fixed in the mixed models, and BLUEs were computed. For image-based traits, BLUEs were rescaled to 0–1 using min–max normalization [68]:


(4) \begin{equation*} f(v)= \frac{v-min(v)}{max(v)-min(v)}, \end{equation*}

where *f(v)* is the normalized value, and min(*v*) and max(*v*) are the minimum and maximum observed values, respectively.

PCA and pairwise Pearson correlations among traits were evaluated using BLUEs. These analyses were conducted in R v4.1.2 [64] with the pheatmap v1.0.12 [69] and ggplot2 v3.5.1 [70] packages for visualization. For PCA, all BLUEs were rescaled using the same min–max normalization method described above.

### Genotyping

DNA extraction was performed by Intertek ScanBi Diagnostics (Alnarp, Sweden) using leaf samples from the 256-genotype population. Genotyping was conducted by Diversity Arrays Technology Pty Ltd (ACT, Australia) using the DArTag method, a targeted genotyping-by-sequencing (GBS) approach validated by Endelman *et al.* [71]. The GBS assay comprised 2500 SNPs, mostly derived from the SolCAP SNP array (Solanaceae Cooperative Agricultural Project, USDA) and evaluated on individuals from the germplasm collections of the International Potato Center (CIP, Lima, Peru) and the University of Wisconsin (USA), using a minor allele frequency (MAF) of 1%.

Although the overall rate of missing data in the population was low (~0.1%), it was attributed to exclusively three genotypes: chip potato breeding clone 97 (Swedish University of Agricultural Sciences), and the cultivars ‘Leyla’ (released in Germany in 1988) and ‘Red Lady’ (released in Germany in 2004). These genotypes lacked sufficient SNP information and were therefore excluded from the prediction models. The SNPs were encoded as tetraploid allele dosages, with 0 representing the reference homozygous class, 4 the alternative homozygous class, and 1, 2, and 3 representing the heterozygous classes using R v4.1.2 [64]. A total of 194 SNPs with MAF < 0.05 were removed from the dataset. To visualize the population structure, we employed a PCA conducted in R v4.1.2 [64] and visualized through ggplot2 v3.5.1 [70].

### Prediction models

We adopted two main strategies for prediction: (i) feature-based regression, where each SNP or image-based trait was treated as an individual input feature in Bayesian regression and machine learning models, and (ii) kernel-based regression, where SNPs and image-based traits were used to construct kernel matrices for use in Bayesian linear mixed-effects models.

#### Feature-based regression

In the feature-based regression approach, each SNP (from the genomics layer) or each image-derived trait (from the phenomics layer) was treated as an independent input feature. For genomics data, the prediction target was the set of BLUEs for both conventional and image-based traits. For phenomics data, we predicted only the BLUEs associated with conventional traits. No hyperparameter tuning was performed in any of the models. Thus, any hyperparameters not explicitly stated were left at their default settings in the respective software libraries.

We first evaluated BRR, implemented using the R package BGLR v1.1.0 [72]. The model used can be expressed as [72]:


(5) \begin{align*} y=X\alpha + \epsilon,\end{align*}

where *y* is the vector of BLUEs for the target trait; *X* is the matrix of input features (either SNPs or image-based traits); $\alpha \sim MVN\mathrm{\big(0,}I{\sigma}_{\alpha}^2\big)$ are the regression coefficients, modeled as random effects with variance ${\sigma}_{\alpha}^2$, assigned a scaled-inverse *χ*^2^ prior; and $\epsilon \sim MVN\mathrm{\big(0,}I{\sigma}_{\epsilon}^2\big)$ is the residual term, with variance ${\sigma}_{\epsilon}^2$.

In addition to BRR, we implemented several machine learning algorithms using the Python scikit-learn library v1.6.1 [73]. An MLP neural network was employed with one hidden layer of 100 neurons, using the rectified linear unit (ReLU) as the activation function. The network was trained using the Adam optimizer with a constant learning rate of 0.001, and the loss function minimized was the mean squared error (MSE). In this architecture, each neuron in the hidden layer performs a linear transformation of the input features followed by the ReLU activation, and the output layer aggregates these representations to produce the final prediction.

Two ensemble strategies were employed: random forest (RF) and adaptive boosting (AdaBoost). In both cases, decision tree regressors were used as base learners, with 100 estimators for RF and 50 for AdaBoost. A decision tree regressor builds a set of hierarchical rules that partition the feature space based on splits that reduce the prediction error with respect to the target variable. RF aggregates the predictions of multiple trees trained on bootstrapped samples, while AdaBoost sequentially fits trees, giving higher weight to observations with larger prediction errors. The last model was a support vector machine (SVM) with a radial basis function (RBF) kernel. In this approach, the kernel projects the input data into a higher-dimensional space where linear relationships can better capture complex patterns associated with the target variable.

#### Kernel-based regression

To integrate information from multiple sources, we adopted distinct variance–covariance structures for each omics layer: (i) a genomic relationship matrix *G* for SNP data, and (ii) a Gaussian kernel matrix *P* for phenomics, calculated with the image-based traits. The genomic relationship matrix *G* was computed using the R package AGHmatrix v2.1.0 [74]. Based on the centered SNP matrix *W* for *n* individuals, *G* was calculated as [75]:


(6) \begin{align*} G= \frac{WW^{T}}{tr(WW^{T})/n}, \end{align*}

where each *G*_i,j_ element represents the genomic relationship between individuals *i* and *j*. When *i* = *j* , *G*_i,j_ corresponds to the inbreeding coefficient of individual *i*. The phenomic relationship matrix *P* was computed using a Gaussian kernel with bandwidth parameter set to 1 [76]:


(7) \begin{equation*} P\left({x}_i,{x}_j\right)=\exp \left\{-\frac{1}{p}\sum \limits_{k=\mathrm{1}}^p\Big({x}_{i,k}-{x}_{j,k}{\Big)}^2\right\}, \end{equation*}

where *x_i_* and *x_j_* are vectors of *p* image-derived traits for individuals *i* and *j*, respectively. Each element *P*_*i*,*j*_ thus captures the phenotypic similarity between individuals based on HTP data.

The full linear mixed model combining both omics layers is defined as:


(8) \begin{align*} y= 1_{\eta}\mu+Z_{1}g+Z_{2}p+\epsilon, \end{align*}

iwhere *y* is a vector of BLUEs for a given trait; 1*_n_* is a vector of 1s; *μ* represents the overall population mean; *Z*_1_ is the incidence matrix linking each observation to a corresponding genetic effect ($g\sim MVN\mathrm{\big(0,}G{\sigma}_g^2\big)$, where ${\sigma}_g^2$ is the genetic variance and *G* is the genomic relationship matrix); *Z*_2_ is the incidence matrix linking each observation to the image-based phenotypic effect ($p\sim MVN\mathrm{\big(0,}P{\sigma}_p^2\big)$, where ${\sigma}_p^2$ is the variance associated with image-based traits and *P* is the phenomic kernel matrix); and *U* represents the residual term ($\epsilon \sim MVN\mathrm{\big(0,}I{\sigma}_{\epsilon}^2\big)$, where ${\sigma}_{\epsilon}^2$ is the residual variance and I*_n_* the identity matrix of dimension *n*, assuming independent and identically distributed residuals).

This model was applied in three different scenarios, with *Z*_1_ = *Z*_2_ = *I*_n_: (i) genomic prediction (genomic best linear unbiased prediction, G-BLUP), using only SNP data to predict conventional phenotypes, where $y\sim MVN{\mathrm{\big(1}}_n\mu, G{\sigma}_g^2+I{\sigma}_{\epsilon}^2\big)$; (ii) phenomics-based prediction (phenomic best linear unbiased prediction, P-BLUP), using only image-based traits, where $y\sim MVN{\mathrm{\big(1}}_n\mu, P{\sigma}_p^2+I{\sigma}_{\epsilon}^2\big)$; and (iii) multi-omic prediction (genomic + phenomic best linear unbiased prediction, G + P-BLUP), combining SNP and image data simultaneously, where $y\sim MVN{\mathrm{\big(1}}_n\mu, G{\sigma}_g^2+P{\sigma}_p^2+I{\sigma}_{\epsilon}^2\big)$. In scenarios (i) and (ii), only one random effect was included in the model, depending on the data source used.

Parameter estimation was carried out under a Bayesian framework using the R package BGLR v1.1.0 [72]. The posterior distributions of the unknown parameters were estimated using a Gibbs sampler with 20 000 iteractions and a burn-in period of 2000 cycles. Finally, we applied the multi-omic prediction model to estimate the variance components attributable to genomics, phenomics, and residual effects.

### Cross-validation scenarios and model evaluations

To evaluate the performance of the prediction models, we used two cross-validation (CV) strategies that reflect common plant breeding selection schemes: (i) predicting the genetic merit of untested genotypes within the same environment (CV1) and (ii) predicting the genetic merit of untested genotypes in different environments (CV00). Given the diversity and potential variability in the genetic architecture of the evaluated traits, both strategies were implemented to enable a comprehensive assessment of predictive ability.

Datasets were split according to these scenarios using conventional machine learning procedures implemented in R v4.1.2 [64] and Python’s scikit-learn library v1.6.1 [73]. Initially, we employed a LOO approach, applied separately to each combination of environment and trait. This method runs *n* iterations, where *n* is the number of genotypes. In each round, the model is trained on *n* − 1 genotypes using their SNP data to learn the association with the observed trait BLUEs, and the remaining genotype is used for prediction.

Due to the large number of combinations of traits, prediction models, and CV strategies, we adopted the LOO scheme. Additionally, from a model evaluation standpoint, LOO allows us to assess predictive performance for every genotype in the dataset while maximizing the use of available information during training. This approach is particularly appropriate in our case, as the study is based on a diverse panel rather than on biparental or closely related populations.

After all LOO rounds, predictions are aggregated into a single vector and compared with the observed BLUEs. In CV1, predictions and observations are from the same environment, while in CV00 we compared the predictions with the observed BLUEs from an unseen environment. To evaluate model performance, we applied multiple metrics. The squared Pearson correlation coefficient (*R*^2^) was used to quantify the strength of the linear relationship between observed values ($\hat{y}$) and predicted values ($\hat{y}$). We selected *R*^2^ because it also reflects the proportion of variance explained, commonly interpreted as predictability or validation reliability. This metric was considered a measure of prediction accuracy and was calculated as follows [77]:


(9) \begin{equation*} {R}^2={\left(\frac{\sum \limits_{i=\mathrm{1}}^n\left({y}_i-\overline{y}\right)\left({\hat{y}}_i-\overline{\hat{y}}\right)}{\sqrt{\sum \limits_{i=\mathrm{1}}^n\Big({y}_i-\overline{y}{\Big)}^2}\sqrt{\sum \limits_{i=\mathrm{1}}^n\Big({\hat{y}}_i-\overline{\hat{y}}{\Big)}^2}}\right)}^2, \end{equation*}

where ${y}_i$ and ${\hat{y}}_i$ are the observed and predicted values for the *i*th individual, $\overline{y}$ and $\overline{\hat{y}}$ are their respective means, and *n* is the number of observations. To assess the rank correlation between predicted and observed values, we used Kendall’s tau (*τ*), calculated as [78]:


(10) \begin{equation*} \tau =\frac{C-D}{\frac{1}{2}n\Big(n-\mathrm{1}\mathrm{\Big)}}, \end{equation*}

where *C* and *D* represent the number of concordant and discordant pairs, respectively, and *n* is the total number of pairs. To quantify overall prediction error, we calculated the MSE [77]:


(11) \begin{equation*} MSE=\frac{1}{n}\sum \limits_{i=\mathrm{1}}^n{\left({y}_i-{\hat{y}}_i\right)}^2. \end{equation*}

Due to its squared units, we also included the mean absolute error (MAE) [77]:


(12) \begin{equation*} MAE=\frac{1}{n}\sum \limits_{i=\mathrm{1}}^n\mid{y}_i-{\hat{y}}_i\mid . \end{equation*}

To compare the use of G-BLUP, P-BLUP, and G + P-BLUP, we performed 5-fold cross-validation within each environment, repeated 50 times. This simulates prediction of untested genotypes under the same environmental conditions, but with a smaller training population than in LOO. Compared with LOO, *k*-fold CV better reflects practical breeding scenarios, in which predictions are made for groups of genotypes using models trained on a reduced subset of the population. This strategy also provides insight into the distribution and stability of predictive performance across different data partitions.

Accuracy was assessed using the same metrics described above, and the squared Pearson correlation coefficients *R*^2^ were visualized as boxplots using ggplot2 v3.5.1 [70]. Statistical differences among modeling approaches were assessed using the non-parametric Kruskal–Wallis rank-sum test. When significant differences were detected, pairwise comparisons were performed using the Wilcoxon rank-sum test, with *P*-values adjusted according to the Benjamini–Hochberg procedure to control the false discovery rate. All analyses were conducted in R v4.1.2 [64].

## Supplementary Material

Web_Material_uhag175

## Data Availability

The marker and phenotypic data used in this study are available in Supplementary Data Tables S10 and S11.
